# Validation of a novel Kinect-based device for 3D scanning of the foot plantar surface in weight-bearing

**DOI:** 10.1186/s13047-019-0357-7

**Published:** 2019-09-02

**Authors:** Giulia Rogati, Alberto Leardini, Maurizio Ortolani, Paolo Caravaggi

**Affiliations:** Movement Analysis Laboratory, IRCCS Istituto Ortopedico Rizzoli, Via di Barbiano 1/10, 40136 Bologna, Italy

**Keywords:** Kinect, 3D scanner, Foot plantar surface, Medial longitudinal arch, Weight-bearing, Custom insoles

## Abstract

**Background:**

Advancements in additive manufacturing, along with new 3D scanning tools, are increasingly fulfilling the technological need for custom devices in personalized medicine. In podiatry and in the footwear industry, custom orthotic and footwear solutions are often required to address foot pathologies or morphological alterations which cannot be managed with standard devices. While laser scanners are the current gold-standard for 3D digitization of the foot shape, their costs limit their applications and diffusion, therefore traditional operator-dependent casting methods are still in use. The aim of this study was to design and validate a novel 3D foot scanner based on the Microsoft Kinect sensor, allowing a 3D scan of the plantar shape of the foot to be acquired in weight-bearing.

**Methods:**

The accuracy and repeatability of the prototypal foot scanner were investigated in a population of 14 asymptomatic healthy subjects, with no history of foot or lower limb injuries. The accuracy was estimated by comparing the Kinect foot scans with those obtained with a high-resolution laser scanner used as reference. The repeatability was assessed by comparing scans of the same foot acquired in different sessions.

**Results:**

The inter-subject average Root Mean Square Error (RMSE) of the Kinect scans was lower than 3 mm for the whole plantar surface, and lower than 1.6 mm for the arch region alone, both in left and right feet. The repeatability, quantified as the average RMSE of pairwise comparisons between sessions, was 1.2 ± 0.4 mm.

**Conclusions:**

The present Kinect-based 3D foot scanner showed optimal intra-operator repeatability and its accuracy appears adequate to obtain 3D scans of the foot plantar surface suitable for different clinical applications. This device could represent a valid low-cost alternative to expensive laser-based scanners and could be used for automatic foot measurements, supporting the design of custom insoles and footwear.

## Background

There is an increasing interest in the research and development of new tools for 3D scanning and modelling of body parts to address the requirements of personalized orthotic devices and treatments [1]. In podiatry and in the footwear industry, an accurate geometrical characterization of the foot shape is critical to designing custom orthoses and footwear for different categories of people, from healthy workers [2, 3] and athletes [4], to patients with foot or lower limb issues [5], such as patients with diabetes [6, 7]. The medial longitudinal arch, the most notable morphological feature used to characterize the foot type [8, 9], allows the foot to act like a spring: when loaded vertically, it stores energy in several visco-elastic structures, such as the plantar aponeurosis, that can be recovered through elastic return [10]. The foot shape resulting from the mechanical interaction with footwear and orthoses is revealed in weight-bearing conditions, when body weight and ground reaction forces act to deform the foot joints and soft tissues spanning the medial longitudinal arch. It is widely reported that significant peak pressure reduction can be obtained with custom orthoses with respect to non weight-bearing designs [11], and to off-the-shelf insoles [12, 13]. However, traditional foot casting methods can not always model the foot in weight-bearing, do not provide automatic foot measurements [14], and have been shown to be less reliable than digital scanning [15, 16]. In this respect, structured-light and laser- based scanners are currently the gold-standard for acquiring 3D high spatial-resolution images of the foot shape. In the former, a pattern of light is projected onto the foot, and cameras located at different positions detect the distortion of the pattern [17]. Laser scanners use an emitter to project a laser line on the foot and capture its reflection with one or more sensors. Both scanners use the triangulation process to reconstruct the 3D shape of the foot.

Although 3D scans are significantly cost-effective compared to the consumable costs of plaster casts [18, 19], commercial 3D foot scanners are still rather expensive (6.000–15.000€) thus strongly limiting their applications and diffusion. For this reason, the Kinect sensor, a RGB-depth camera developed by Microsoft for the videogame industry, appears to be a viable low-cost solution for 3D scanning of the foot shape. The sensor has found application as a static body scanner, e.g. in the assessment of postural control [20] and spinal deformity [21], and as a motion analysis tool [22–26]. While the Kinect has been used to analyse foot posture and morphology [27–29], a thorough validation study as a 3D foot scanner has yet to be reported.

The purpose of this study was to validate, in terms of accuracy and repeatability, a novel 3D foot scanner based on the low-cost Microsoft Kinect sensor to obtain 3D images of the foot plantar surface in weight-bearing.

## Methods

**Fig. 1:**
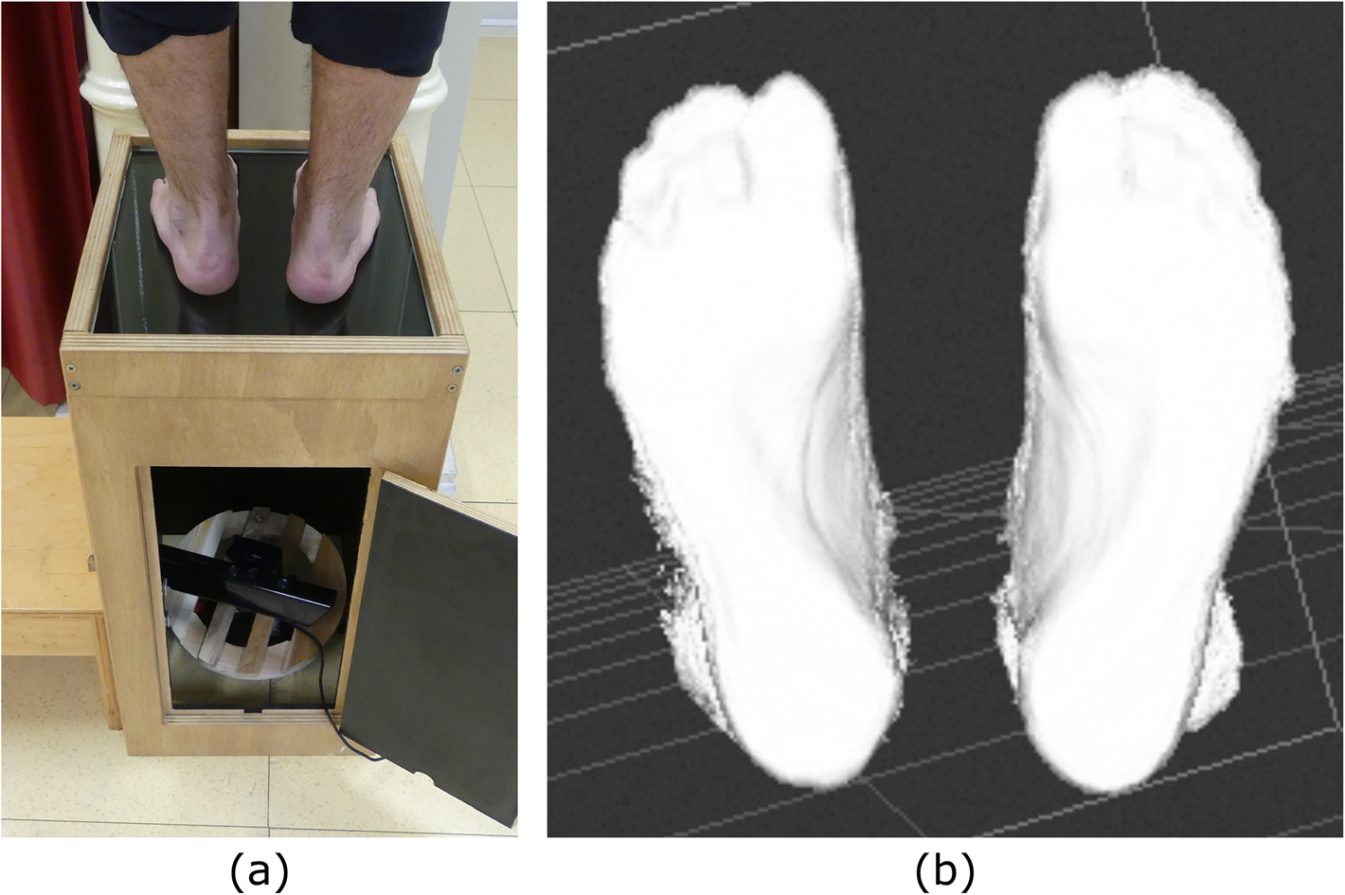
**a** The Kinect-based 3D foot scanner. The Kinect sensor is located on a rotating platform at the bottom of the wood box. On top, a 15 mm thick glass plate allows to scan the foot plantar surface in different loading conditions. **b** 3D point cloud of the plantar foot shape of one subject in bipedal standing, visualised in Skanect

The Kinect sensor is an RGB-depth camera, developed by Microsoft (Redmond, US), which captures simultaneous depth and colour images of the surrounding environment [30]. Initially developed to enhance the gaming experience of the Xbox 360 console, the Kinect sensor has also been employed outside the gaming industry due to the release of a Software Development Kit running on MS Windows. In this investigation, a Kinect-based (Kinect for Windows, 2012 version) foot scanner was designed and tested for accuracy and repeatability. The Kinect sensor combines a laser emitter, an infrared and an RGB camera to obtain a 300.000 point-cloud 3D image of the scanned object via triangulation process [31], at a maximum of 30 fps. The scanner consists of a 0.82 m tall wood box, comprising a scanning glass plate at the top and the Kinect sensor at the bottom (Fig. 1a). The sensor is positioned above a rotating plate, manually rotated during scanning, which allows the plantar aspect of the foot to be visualized from different angles, while preserving the focus distance at 0.55 m. The scanning plate consists in a 400 × 350 × 15 mm glass plate allowing the foot plantar surface to be visualised by the depth sensor. In order to minimize light reflections, the interior walls of the wood box are painted in black and external light sources were reduced during scanning. The acquisition time was set to 25 s, to allow a slow 360 deg. rotation of the sensor. The software Skanect for Windows (Skanect by Occipital, version 1.8) was used to acquire and pre-process the raw 3D depth-data of the scanned feet (Fig. 1b). The laptop computer used for data acquisition (Intel Core i5 6300 HQ @2.30 GHz, 12GHz RAM) allowed to acquire high-quality 3D images up to 21 fps.

**Fig. 2:**
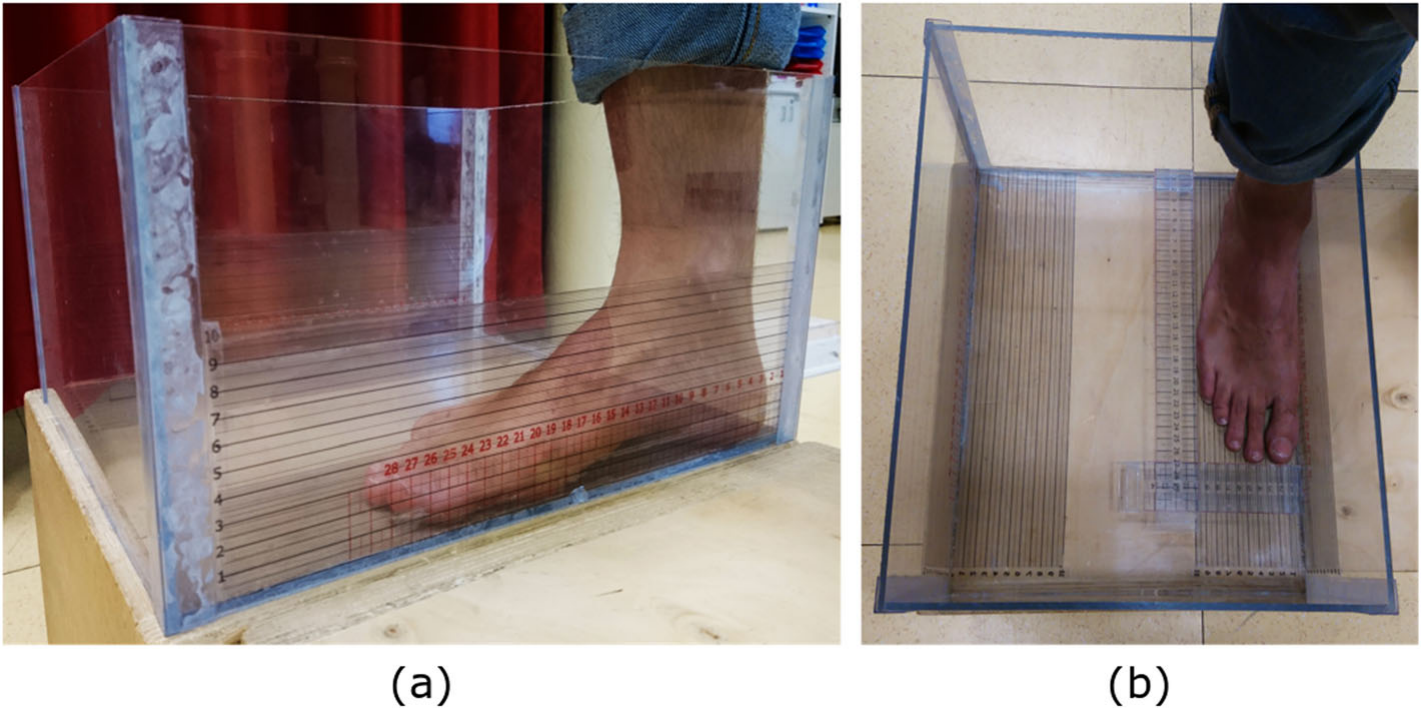
The PodoBox: a plexiglass-made foot measuring tool. Adhesive rulers on the sides and bottom surface for arch height and arch length measurement (**a**), and transparent mobile rulers for foot length and foot width measurement (**b**)

Fourteen asymptomatic healthy volunteers (age 21–61 years; BMI 23 ± 3 kg/m^2^; shoe size 37–43 EU) with no history of foot and lower limb trauma or surgery were recruited for this study. According to the podoscopic evaluation the subjects were classified as having slightly flat feet (*n* = 5), rectus feet (*n* = 6) and slightly cavus feet (*n* = 3). A plexiglass box for foot measurements (PodoBox), featuring transparent rulers on the sides and in the bottom surface, was used to measure the foot main morphological parameters (Fig. 2). The accuracy of the device was assessed by comparing the scans of 28 feet from 14 subjects with those obtained with a high-resolution commercial 3D foot scanner (i-Qube, Delcam, UK). The scans were taken in bipedal standing - or full weight-bearing; this posture was preferred over other weight-bearing conditions for it is highly repeatable, thus helping to reduce differences in the foot shape on the two scanning devices. The 3D modelling software Geomagic Control™ (3D Systems, Rock Hill, USA) was used for spatial alignment and to calculate the average distance between the Kinect and i-Qube scans. The alignment was achieved via the “Best Fit Alignment” procedure: a preliminary gross alignment is performed by matching 5000 random points, and finer adjustments can be obtained by using 25.000 random points, until the average deviation between scans is minimized [32]. Distance Maps - i.e. the point-by-point graphical representation of the distance between the two 3D data sets - and the Root Mean Square Error (RMSE) of the distances were used to quantify the accuracy of the Kinect scans in the whole plantar surface and in the medial arch region alone. The latter was investigated independently because of its relevance to foot type classification and custom orthotics design.

The repeatability of the Kinect scans across different sessions was also assessed on 28 feet of 14 subjects. The feet of each subject were scanned in three sessions a few days apart, and pairwise comparisons were performed to calculate the RMSE between scans of the same foot. Three RMSE were therefore calculated, for both right and left foot of each subject - session#1 vs. session#2, session#1 vs. session#3 and session#2 vs. session#3 - and the average RMSE for each subject’s foot was averaged across all subjects. The Coefficient of Variation of the RMSE distribution across comparisons was also used to assess the scans repeatability.

**Fig. 3:**
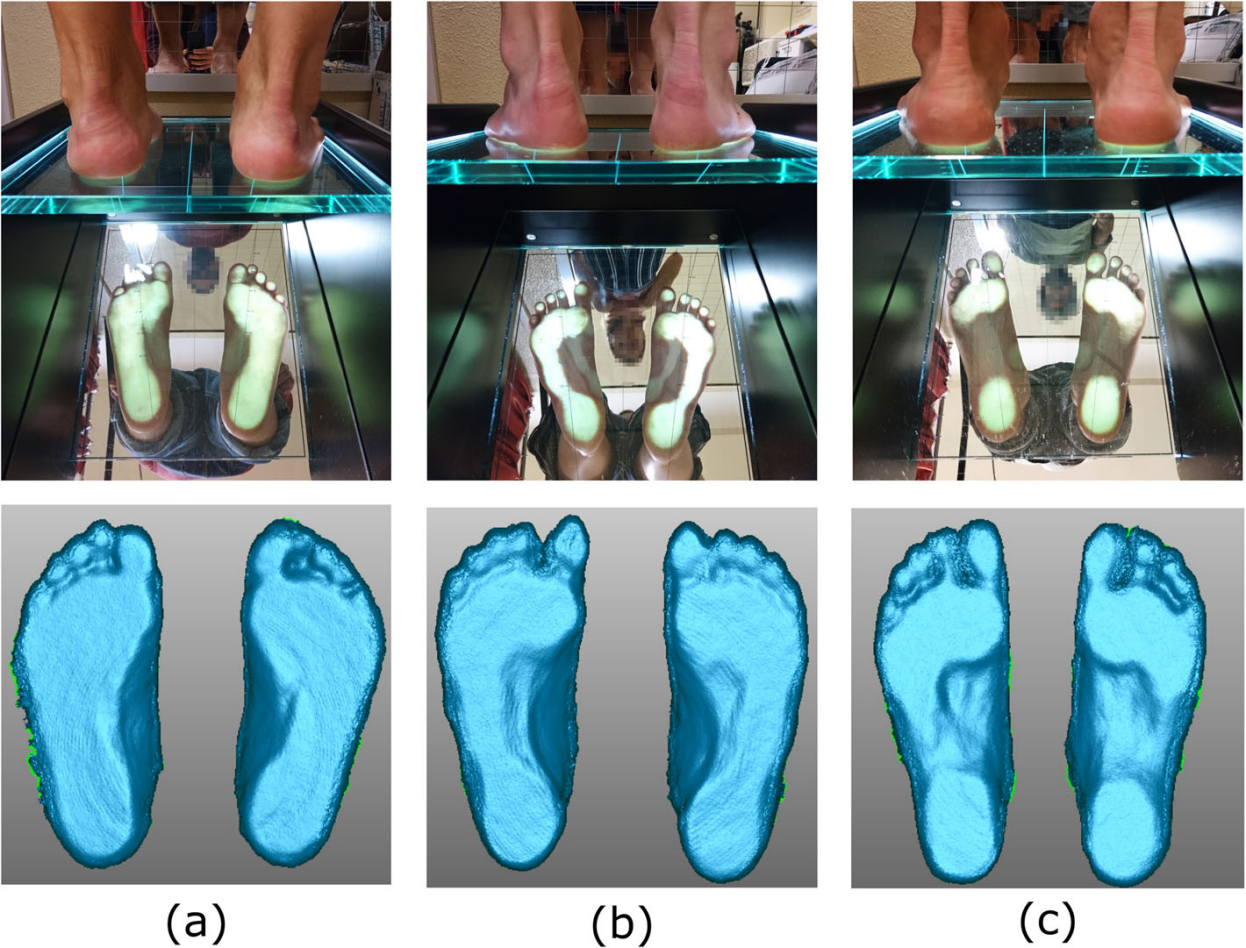
Sample flat (**a**), rectus (**b**) and cavus (**c**) feet scanned in bipedal standing posture. Top, podoscope visualization; bottom, corresponding Kinect scans displayed in Geomagic

In order to assess the scanner effectiveness in detecting differences between foot types, the main morphological parameters of six sample subjects - two with flat, two with rectus and two with cavus feet - were analysed (Fig. 3). Custom software was developed in Matlab (MathWorks, R2016a) for the automatic analysis of the Kinect foot scans to estimate foot length, foot width, arch height, arch width, arch length and the Arch Index. The arch width refers to the maximum penetration of the foot arch in the medio-lateral direction, expressed as a percentage of the foot width at the same position. The Arch Index is computed as the ratio between the area of the middle third of the footprint and the total area of the footprint - toes excluded [33]. Furthermore, manual measurements of foot length and foot width, obtained using the PodoBox, were compared to the corresponding estimated by the software on the 3D scans, and the average % errors were computed.

## Results

### Kinect-scans accuracy

**Fig. 4:**
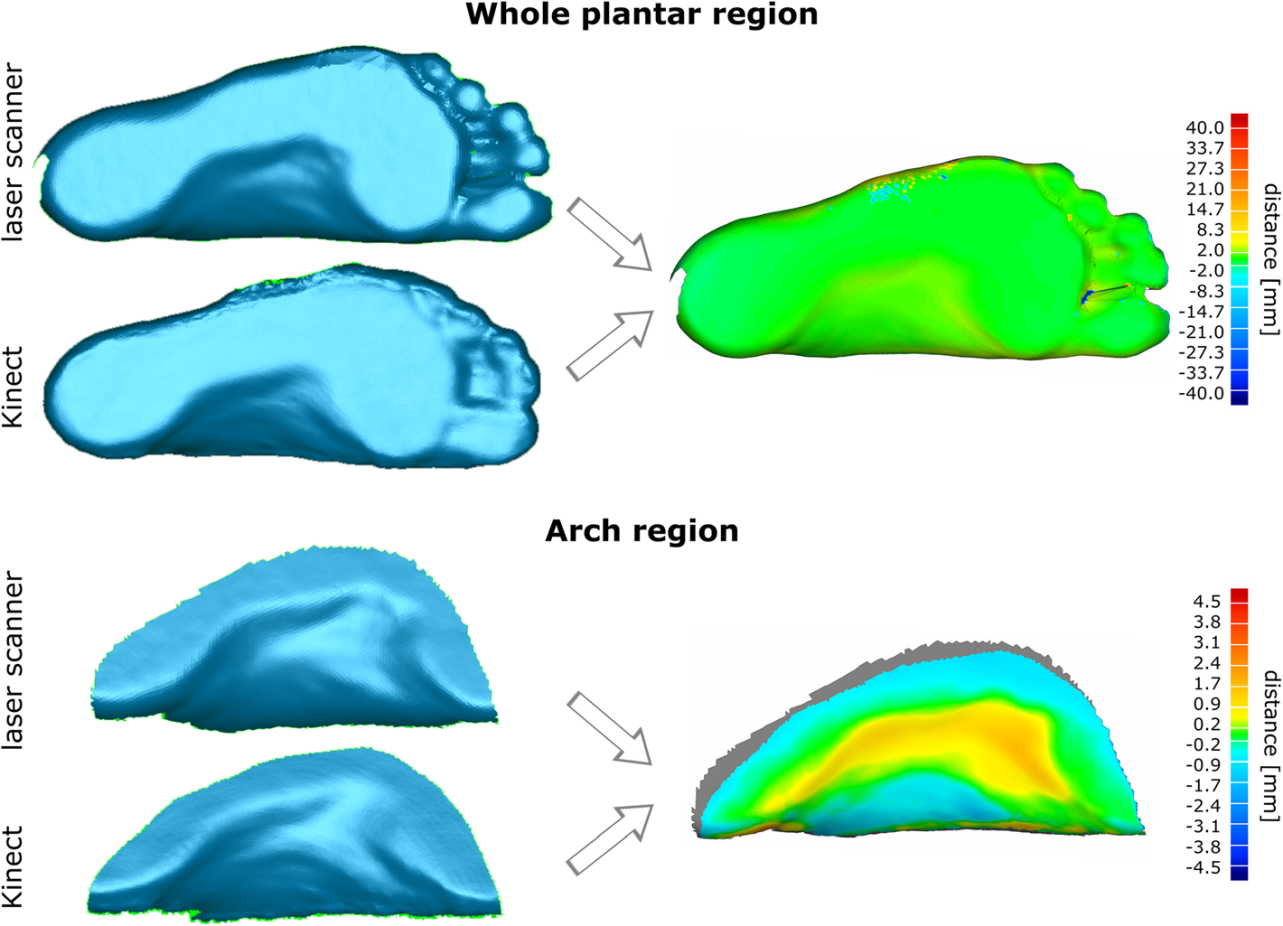
Color maps of the distances [mm] between laser scanner and Kinect foot scans for the right foot of one subject in weight-bearing, following the Geomagic ^“^Best Fit Alignment” procedure. Top, error in the whole plantar region; bottom, error in the arch region alone

The accuracy in the 3D scan of the arch region was higher than that of the whole plantar surface. The comparison between 3D scans of the whole plantar surface obtained with the Kinect and with the high-resolution laser scanner (Fig. 4, top), resulted in an inter-subject average RMSE of 2.8 ± 0.6 mm and 2.9 ± 0.4 mm, respectively across left and right feet. Corresponding errors in the arch region alone (Fig. 4, bottom) were 1.4 ± 0.4 mm and 1.6 ± 0.5 mm (Table 1).

**Table 1:** RMSE [mm] between Kinect-based and reference foot scans for each left and right foot of the 14 subjects. The inter-subject average RMSE is reported at the bottom of the table

Subject ID	RMSE [mm]			
	Left foot whole plantar surface	Right foot whole plantar surface	Left foot arch region	Right foot arch region
Subj 01	2.8	3.8	1.8	1.6
Subj 02	2.2	2.6	1.5	2.3
Subj 03	2.8	3.2	1.8	0.7
Subj 04	3.3	2.7	0.7	0.9
Subj 05	3.5	2.9	1.5	2.0
Subj 06	2.3	2.2	1.6	1.8
Subj 07	2.9	3.6	1.3	1.3
Subj 08	3.5	2.2	1.0	2.5
Subj 09	2.7	3.5	1.5	1.2
Subj 10	3.2	2.8	0.9	1.5
Subj 11	1.6	1.9	1.1	1.1
Subj 12	2.9	3.3	2.0	1.9
Subj 13	2.5	3.7	1.4	1.5
Subj 14	3.6	2.3	1.6	2.2
Mean ± SD	2.8 ± 0.6	2.9 ± 0.4	1.4 ± 0.4	1.6 ± 0.5

### Kinect-scans repeatability

Good repeatability of the Kinect scans was observed across all trials. The inter-subject average RMSE of the pairwise comparisons between scans acquired in three sessions was 1.2 ± 0.4 mm, both for left and right feet (Table 2). The inter-subject average Coefficient of Variation was 24.3 ± 12.3% and 17.4 ± 10.5%, respectively across left and right feet.

**Table 2:** RMSE [mm] of the distances calculated in the pairwise comparisons between the three scans of each left and right foot of the 14 subjects

Subject ID	RMSE [mm] - Left foot whole plantar surface				RMSE [mm] - Right foot whole plantar surface			
	trial1 vs trial2	trial1 vs trial3	trial2 vs trial3	mean ± SD	trial1 vs trial2	trial1 vs trial3	trial2 vs trial3	mean ± SD
Subj 01	1.1	1.1	0.8	1.0 ± 0.2	1.2	1.4	1.1	1.2 ± 0.1
Subj 02	1.3	1.4	0.7	1.1 ± 0.4	0.9	0.8	0.5	0.8 ± 0.2
Subj 03	1.2	1.0	0.6	0.9 ± 0.3	1.4	1.4	1.5	1.4 ± 0.1
Subj 04	1.6	1.6	0.8	1.3 ± 0.5	1.3	1.5	1.2	1.3 ± 0.2
Subj 05	1.0	0.9	1.0	1.0 ± 0.0	0.8	0.8	0.5	0.7 ± 0.2
Subj 06	1.3	1.8	1.0	1.3 ± 0.4	1.9	2.0	1.0	1.7 ± 0.5
Subj 07	1.8	1.7	0.9	1.5 ± 0.5	1.8	1.6	0.9	1.4 ± 0.5
Subj 08	0.9	1.0	1.3	1.1 ± 0.2	1.7	1.8	1.1	1.5 ± 0.4
Subj 09	2.2	1.9	0.9	1.7 ± 0.7	1.2	1.1	1.0	1.1 ± 0.1
Subj 10	1.2	1.3	1.1	1.2 ± 0.1	1.2	1.2	0.9	1.1 ± 0.1
Subj 11	1.2	1.5	1.4	1.4 ± 0.2	1.3	1.3	1.2	1.0 ± 0.0
Subj 12	0.9	0.6	0.7	0.7 ± 0.2	1.0	0.9	0.9	1.1 ± 0.1
Subj 13	0.9	0.8	0.7	0.8 ± 0.1	1.2	1.0	1.2	0.9 ± 0.1
Subj 14	1.0	1.0	0.5	0.9 ± 0.3	1.0	1.1	0.7	1.0 ± 0.2

### Morphological parameters of sample flat, rectus and cavus feet

The automatic analysis of the foot scans allowed estimation of the foot main morphological parameters for the 12 feet of the six sample subjects (Table 3). The main foot dimensions, foot length and foot width, were similar to the corresponding PodoBox measurements: the inter-subject average error was 1.2 ± 1.1% and 0.9 ± 0.7%, in foot length and 9.0 ± 4.1% and 10.2 ± 3.2% in foot width, respectively across left and right feet. Arch Index and arch width values were consistent with the clinical classification; the largest Arch Index and lowest arch width were found for the four flat feet, whereas the lowest Arch Index and the largest arch width were found for the four cavus feet.

**Table 3:** Morphological parameters of the 12 feet clinically classified as Flat [2], Rectus [2] and Cavus [2]: Arch Index, foot length, foot width, arch height, arch width and arch length

Foot type		Arch Index	Foot length [*mm*]	Foot width [*mm*]	Arch height [*mm*]	Arch height *[% foot length]*	Arch width *[% width]*	Arch length [*mm*]	Arch length *[% foot length]*
flat #1	Left	0.33	268.1	105.6	12.5	4.7	36.4	122.0	45.5
	Right	0.31	266.4	102.5	9.8	3.7	33.3	82.0	30.8
flat #2	Left	0.30	259.4	114.3	18.8	7.2	36.4	78.0	30.1
	Right	0.29	258.9	117.5	18.9	7.3	31.8	82.0	31.7
rectus #1	Left	0.27	271.9	111.0	23.6	8.7	50.0	90.0	33.1
	Right	0.26	268.0	113.1	20.2	7.5	46.0	76.0	28.4
rectus #2	Left	0.26	244.9	101.4	24.5	10.0	56.8	80.0	32.7
	Right	0.27	239.3	103.8	17.2	7.2	52.2	82.0	34.3
cavus #1	Left	0.10	243.0	96.7	17.9	7.4	100.0	104.0	42.8
	Right	0.05	243.5	101.4	17.9	7.3	100.0	88.0	36.1
cavus #2	Left	0.07	269.5	99.2	15.6	5.8	100.0	104.0	38.6
	Right	0.12	268.9	102.1	19.6	7.3	100.0	86.0	32.0

## Discussion

In podiatry, foot impression foams are still the preferred method to design semi weight-bearing custom insoles. Most of the current optical and laser-based foot scanning devices are either expensive for small clinics and non-commercial applications, or do not allow foot scanning in weight-bearing. Moreover, no automatic tool is currently available to estimate the foot’s main morphological parameters from 3D foot scans. The advent of depth sensors, associated to infrared cameras and projectors, represent a cost-effective scanning tool with respect to laser-based devices. The purpose of this study was to assess the accuracy and the reliability of a novel low-cost 3D foot scanner based on the Microsoft Kinect sensor.

Preliminary tests were performed to choose the optimal scanning parameters, with the subject in bipedal standing on the scanning plate. A 360 deg. rotation of the Kinect sensor was chosen as the optimal method to guarantee the best quality of the plantar foot scans, and was preferred to a fixed position or to sensor translation.

The accuracy of the Kinect-based foot scanner was assessed on 14 subjects, using a commercial high-resolution 3D laser scanner as reference. The inter-subject average RMSE in the 3D shape of the whole plantar surface was about 3 mm, and this was very similar in the left and the right foot. The largest errors were located at the toes and in the lateral aspect of the midfoot region, approximately along the fifth metatarsal bone (see Fig. 4). It should however be highlighted that some differences in foot posture were expected especially at the toes region, as Kinect-based and reference foot scans could not be acquired simultaneously and thus may be affected by small postural differences. Therefore, the error in scanning the arch region alone, which is the foot’s most prominent morphological feature, was also investigated. The inter-subject average RMSE in the 3D shape of the medial arch was about 1.5 mm, and very consistent between left and right foot. This error, albeit rather small and consistent with the repeatability of the measurements, should be accounted for when designing personalized orthoses or in the statistical comparison of morphological data from different groups.

While the novel scanner showed very good inter-session repeatability (range 0.5 ÷ 2.2 mm), the inter-operator repeatability was not investigated in the present study. However, since the acquisition process is almost fully automatic, inter-operator errors are expected to have similar magnitude.

Further geometrical analysis of the plantar foot scans via custom Matlab scripts showed good agreement between the acquired 3D data sets and the clinical observations. In particular, the estimated Arch Index of the flat feet was larger than that of the cavus feet, while the arch width was the smallest in the flat feet and the largest in the cavus feet. The morphological measurements based on the Kinect foot scans appeared consistent with the real foot morphology and may represent a useful objective tool, in addition to the clinical evaluation, for foot type classification. While the average error in measuring foot length was approximately 1%, the scan-based foot width measurements were about 10% larger than the corresponding measurement from the PodoBox. This small overestimation of the real foot width may be explained by the compression of the soft tissues between the mobile ruler and the side of the PodoBox, compression that was not present during the Kinect scanning (see Fig. 2b).

From an economical perspective, the total cost of the present prototypal 3D foot scanner is about 200–300€, which is at least one order of magnitude lower than that of commercial laser-based foot scanners currently available. This should also allow clinics and research groups with limited resources to perform foot scans to support objective diagnosis of foot pathologies. Moreover, the non-invasive and automatic procedure proposed here allows foot scanning in different loading conditions, and the operator’s influence is minimal. However, the present study and its outcome should be considered in light of some limitations. While accuracy and repeatability results were largely consistent across subjects, and between left and right foot intra-subject, the sample of feet analysed is rather small. In addition, only adult feet were included, thus no information is available on the applicability and reliability of the device in scanning children’s feet. Finally, all acquisitions were performed in ideal low-light conditions with a rather powerful portable computer; lower resolution scans could be obtained and some noise may be present in real-case scenarios.

## Conclusions

This study has shown that the accuracy of the Kinect sensor, within the setup specifically designed for this investigation, is comparable to that of laser-scanner devices. The sensor, therefore, may be reasonably used to obtain 3D scans of the foot plantar surface suitable for different clinical and biomechanical applications. The novel low-cost foot scanner may represent a valid alternative to more expensive laser scanners currently on the market and suitable to support the design of custom insoles and orthoses. The scans geometry can be further analysed and measured by specific software applications, as shown in the present manuscript. The combination of the Kinect-based foot scanner and of relevant analysing code presented here may represent a useful tool for automatic foot measurement, providing podiatrists and clinicians with quantitative parameters for foot type classification and diagnosis of foot morphological alterations.

## Data Availability

The datasets used and/or analysed during the current study are available from the corresponding author on reasonable request.
